# Using of Amniotic Membrane Derivatives for the Treatment of Chronic Wounds

**DOI:** 10.3390/membranes11120941

**Published:** 2021-11-29

**Authors:** Iveta Schmiedova, Alena Dembickaja, Ludmila Kiselakova, Beata Nowakova, Petr Slama

**Affiliations:** 1 BioHealing s.r.o., Slabihoudka 6232/11, 708 00 Ostrava, Czech Republic; iveta.schmiedova@natic.cz (A.D.); ludmila.kiselakova@biohealingeurope.eu (L.K.); 2 National Cell and Tissue Centre, Slabihoudka 6232/11, 708 00 Ostrava, Czech Republic; beata.nowakova@natic.cz; 3 Department of Animal Morphology, Physiology and Genetics, Faculty of AgriSciences, Mendel University in Brno, Zemedelska 1, 613 00 Brno, Czech Republic; petr.slama@mendelu.cz

**Keywords:** amniotic membrane, wound healing, amniochorionic membrane

## Abstract

Amniotic membrane grafts have some therapeutic potential for wounds healing. Early application of amniotic membrane turned out as beneficial in healing ulcers, burns, and dermal injuries. Since the second half of the 20th century, the autotransplants of amniotic/chorion tissue have been also used for the treatment of chronic neuropathic wounds, cornea surface injuries, pterygium and conjunctivochalasis, and dental and neurosurgical applications. The aim of this publication is to prepare a coherent overview of amniotic membrane derivatives use in the field of wound healing and also its efficacy. In total 60 publications and 39 posters from 2000–2020 were examined. In these examined publications of case studies with known study results was an assemblage of 1141 patients, and from this assemblage 977 were successfully cured. In case of posters, the assemblage is 570 patients and 513 successfully cured. From the investigated data it is clear that the treatment efficacy is very high—86% and 90%, respectively. Based on this information the use of the amniotic membrane for chronic wounds can be considered highly effective.

## 1. Introduction

The amniotic membrane is the inner layer of the placenta that encircles the embryo and forms a sac filled with amniotic fluid that surrounds the embryo. The membrane is rich in collagen, thin, transparent, and strong, lining the chorionic layer. The main function of the amniotic membrane is to protect the fetus from unwanted external factors during intrauterine development [1].

The amniotic membrane (amnion) occurs in the prenatal period of human development. It emerges very early, the 7th day after the conception of the oocyte, inside the embryoblast. Embryoblast is firstly divided into two layers, epiblast and hypoblast. The epiblast emerges from the amniotic cavity, that is the cavity surrounded by amnioblasts. During the 4th week of pregnancy, the amnion surrounds almost the whole embryo and the amniotic wall gets in the embryo in the location where the umbilical cord will develop. Inside the amniotic cavity is amniotic fluid [2].

**Figure 1:**
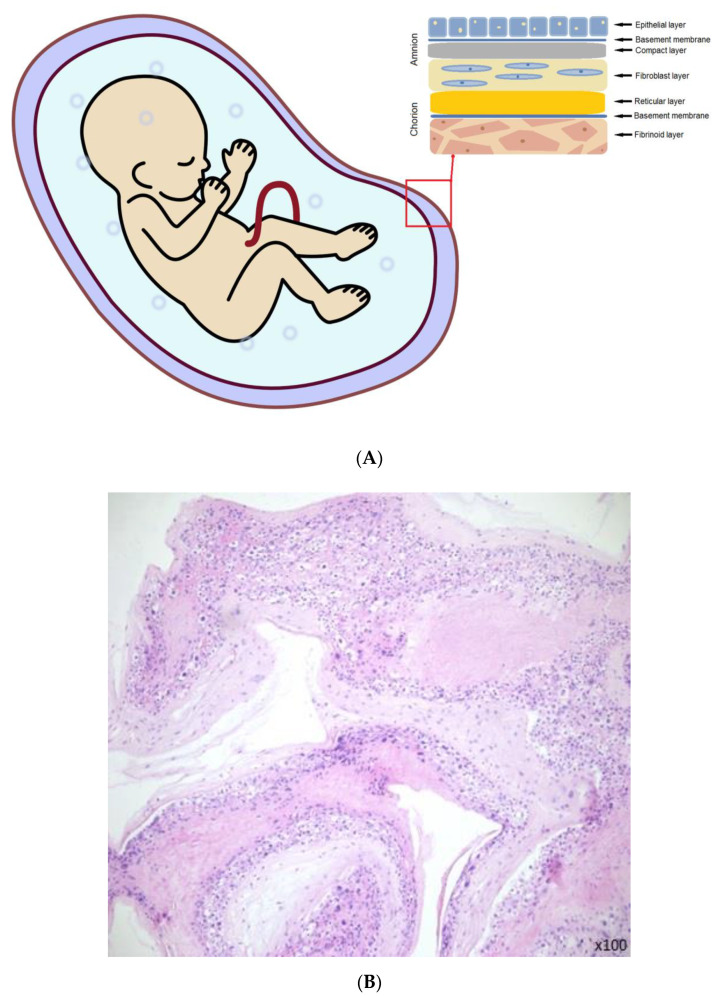
(**A**) The amniotic membrane layers [6]. (**B**) Section of fresh amniotic membrane with epithelial cells.

The amnion is an elastic and semi-permeable tissue made from 5 layers (Figure 1A); the most inner layer, nearest to the fetus and in direct contact with the amniotic fluid are epithelial cells (Figure 1B). These cells have certain characteristics that make them a considerable source of stem cells [3]. Then the thick basal membrane, which main components include laminins, type IV and VII collagen, and fibronectin. Next the stroma, comprised of three layers: the compact layer, fibroblast layer, and the last one which is a spongy layer that fits closely to the surrounding chorion [4]. The outer fibroblast layer contains mesenchymal cells and with the spongy layer makes up most of the thickness of the amnion tissue [5]. Layers secure many functions such as the synthesis of growth factors and cytokines. It is responsible for pH regulation, water transport, and also serves as a semipermeable barrier for amniotic molecules [6].

The fibroblast-like outer mesenchymal layer, which probably originates from the mesodermal embryonic plate, is dispersed in a full-fledged membrane. The layer adjacent to the chorionic laeve has an almost acellular structure and contains a non-fibrillar meshwork of predominantly type III collagen [7]. The fundamental mechanism of the therapeutic effect has not been fully undiscovered yet. The native human amniotic membrane contains plenty of growth factors including the epidermal growth factor (EGF), basal fibroblast growth factor (bFGF), keratinocyte growth factor, transforming growth (TGFa and TGFp), nerve growth factor, and hepatocyte growth factor [8,9], which are well-known for their crucial role in physiological processes leading to normal wound healing and tissue regeneration. Furthermore, it is a suitable natural scaffold for cell proliferation, and/or differentiation. Therefore, scaffolds based on an amniotic membrane have been developed to improve its healing capacity [10]. Due to these properties, the amniotic membrane has been used for clinical applications. In the last 50 years the autotransplants of the amniotic/chorion tissue have been successfully used also for chronic neuropathic wounds, cornea surface injuries, pterygium, conjunctivochalasis, and dental and neurosurgical applications [11].

Regenerative qualities of amniotic membrane

- Analgesic effect: Amniotic membrane covers loose nerve twigs in a wound and reduces the concentration of anti-inflammatory and algic cytokines and peptides which significantly reduces pain in the wound spot.
- Reducing scarring: One of the amniotic membrane’s surfaces is non-adhesive, prevents from growing, and reduces occurring of undesired accretions and fibrotization. The hyaluronic acid present in the amniotic membrane also inhibits excessive fibrotization.
- Epithelization: Amniotic membrane contains tens of types of growth factors many of which directly and significantly supports epithelization. It especially contains epidermal growth factor (EGF), keratinocyte growth factor (KGF), and hepatocyte growth factor (HGF) which support and activate migration, proliferation, and differentiation of epithelial cells.
- Angiogenic effect: Amniotic membrane releases a range of angiogenic factors into the wound, especially bFGB (Fibroblast Growth Factor-basic), TGF-ß (Transforming Growth Factor-beta) which support vessel renewal in the area of the healing wound. Neoagiogenesis augmented by the amniotic membrane significantly shortens the time to regenerate.
- Anti-inflammatory effect: Amniotic membrane contains and releases Interleukin 10 (IL-10) which has the major anti-inflammatory effect, and thrombospondin-1, both are antagonists of the Interleukin 1 (IL-1) receptor and tissue inhibitors metalloproteinases (TIMPs).
- Mechanical effect: Amniotic membrane significantly reduces wound desiccation, and functions as a mechanical support and structure which allows epithelial and mesenchymal cells attachment, motility, and proliferation.
- Amniotic membrane is non-immunogenic: Amnion does not express transplantation antigens (HLA-A, B, C) and does not induce the recipient’s organism’s immune response [12,13,14].

## 2. Clinical Application of the Amniotic Membrane

The amniotic membrane can be used in many various fields of medicine, especially in the treatment of skin burns and prevention of tissue adhesion in surgery of the head, neck, abdomen, larynx, and genitourinary tract [7].

The first report of skin transplantation with the use of the fetal membrane was in 1910 by Davis, J. W., and in 1940, De Roth first reported the use of fetal membranes in the ocular surface [15].

**Figure 2:**
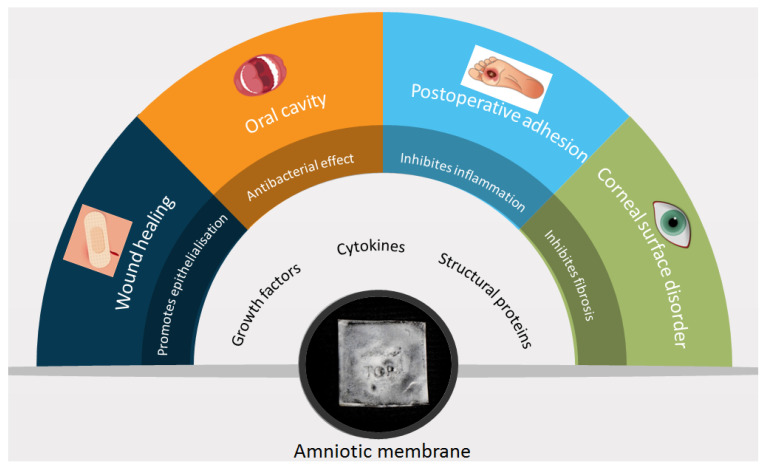
Amniotic membrane components, characteristics, and applications; data collected from cited articles.

**Figure 3:**
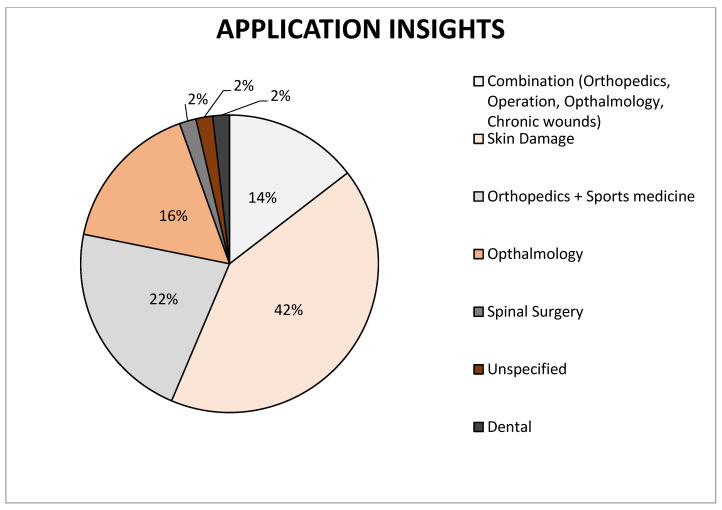
Representation of individual types of amniotic membrane application on the market, data collected from cited articles.

Since then, it has gained popularity as a biologic dressing and/or scaffold for tissue regeneration. The amniotic membrane was used for numerous applications as a surgical dressing for burns and as an adjunctive tissue in surgical reconstruction of the oral cavity, bladder, and also for tympanoplasty, arthroplasty, repair of omphaloceles, and prevention of adhesions in pelvic and abdominal surgery (Figure 2 and Figure 3). All of these applications are the outcome of the interesting features of the amniotic membrane, for example the antimicrobial characteristic has made it a suitable choice for postsurgery applications in wound healing, burn injuries, dental injuries, and ophthalmology as bacterial infection and biofilm growth are regular in these sites [15,16].

Another property of AM (Amniotic membrane) is biocompatibility or the ability not to produce a toxic, injurious, carcinogenic, or immunological response in living tissue, and if it’s not destroyed by inflammation should be able to react to the appropriate host response. In addition, no less important is it that the membrane be biodegradable, making it a suitable choice for use as a scaffold [17]. The use of amniotic membranes for corneal burns and other ophthalmological epithelial defects is common and has led to the development of several commercially available products [12]. Furthermore, the amniotic membrane can be used as an alternative source of stem cells and some studies suggest the use of these cells in tissue repairs, such as corneal tissue, spinal cord injury, brain infarction, and Parkinson’s disease [18].

The AM is a biologically derived material, and there are the same problems as with other applications of biological material. For example, the transmission of infectious diseases is always a risk associated with human organ and tissue transplantation. Therefore, when applying AM, there must be followed the same safety precautions and safety criteria applied to organ transplantation. Potential donors need to be effectively screened for any risk factors that could make them unsuitable for donation such as HIV(Human Immunodeficiency Virus), hepatitis B, hepatitis C, CMV (Cytomegalovirus), syphilis, and other possible infections so that there is little chance that the donor may be in a window period of infection. Even if the serological tests are negative, it is advisable to repeat the screening after 6 months. The AM can be preserved at −80 °C until all test samples are collected and are negative [3,17,19].

**Figure 4:**
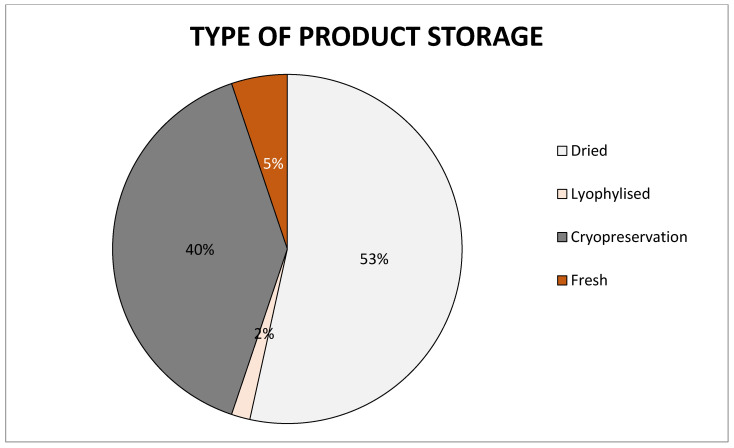
Chart of preservation types of amniotic membrane on the market, data collected from cited articles.

There are several basic methods for storing human AM: cryopreservation, lyophilization, and dry storage. Each of them has its advantages and disadvantages and a different length of storage time (Figure 4).

## 3. Materials and Methods

For the chosen research activity there was chosen an assemblage of 60 publications and 39 posters from 2000–2020. The majority are publications dealing with the case studies about amniotic membrane use for chronic wound healing. Due to the diverse quality of the research assemblage, there were chosen several parameters to be assessed primarily for each publication or poster. These parameters are the number of patients in the study and treated by the amniotic membrane derivatives, the number of patients assessed in the study as successfully cured after the treatment, the type of derivative used (dHAM—derivatives of human amniotic membrane, dHACM—derivatives of human amniochorion membrane, other derivatives-specified), the number of derivative applications and their frequency (weekly/biweekly), the primary diagnosis requiring the treatment and place where the derivatives were applied, the size of the wound (in cm^2^ ± standard deviation), and the time measured in weeks until the wound was cured (in case of the studies with more patients the meantime of cure). The records of the publications and posters are not always complete because of the different structure of the publications and given or concealed information about the process of the studies and treatment.

## 4. Results

**Figure 5:**
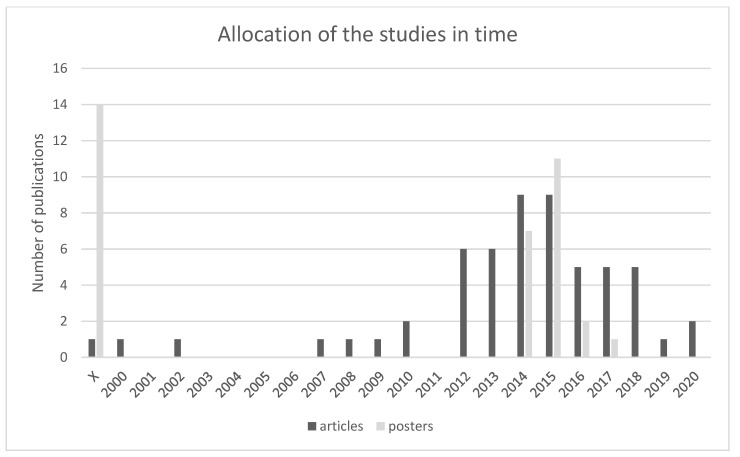
Shows the allocation of publications in time which suggests a significant increase of such publications since 2012. In total, 60 articles and 39 posters were included in the research. Most articles were published between 2014 and 2015 and most posters were published in 2015 [1,2,3,4,5,6,7,8,9,10,11,12,13,14,15,16,17,18,19,20,21,22,23,24,25,26,27,28,29,30,31,32,33,34,35,36,37,38,39,40,41,42,43,44,45,46,47,48,49,50,51,52,53,54,55,56,57,58,59,60,61,62,63,64,65,66,67,68,69,70,71,72,73,74,75,76,77,78,79,80,81,82,83,84,85,86,87,88,89,90,91,92,93,94,95,96,97,98,99].

Duplicate sources were identified in the given research set. As far as articles are concerned, 3 articles were duplicate [20,21], and as far as posters are concerned, one poster was duplicate [22]. Since 2012 there is a notable increase in publications, see Figure 5. The number of articles starts to increase in 2014–2015 and then rapidly decreases until the year 2020. Posters on this topic began to be published after 2014. The largest increase was in 2015, but a year later there was a significant decrease in the number of published posters, and from 2018 to 2020, none were published.

**Figure 6:**
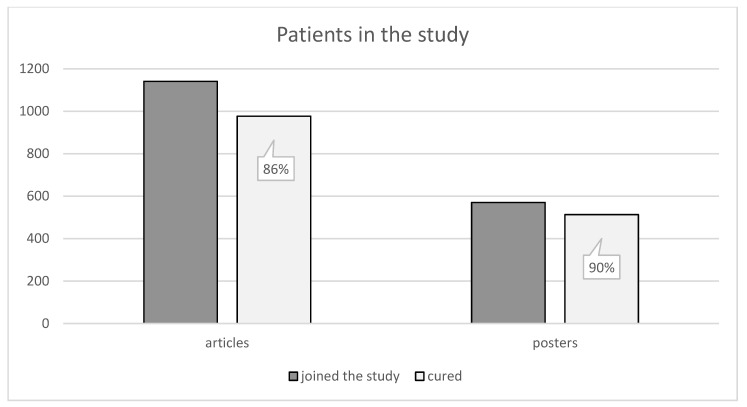
Shows the number of patients. There were 1141 patients included in the articles, 977 of whom have been successfully cured. Posters included 570 patients in total, 513 of whom have been successfully cured [1,2,3,4,5,6,7,8,9,10,11,12,13,14,15,16,17,18,19,20,21,22,23,24,25,26,27,28,29,30,31,32,33,34,35,36,37,38,39,40,41,42,43,44,45,46,47,48,49,50,51,52,53,54,55,56,57,58,59,60,61,62,63,64,65,66,67,68,69,70,71,72,73,74,75,76,77,78,79,80,81,82,83,84,85,86,87,88,89,90,91,92,93,94,95,96,97,98,99].

Within the processed articles from case studies with known treatment results we have covered a set of 1141 patients, 977 of whom have been successfully cured. As far as posters are concerned, 570 patients were involved, 513 of whom have been successfully cured. The high effectiveness of the treatment is apparent from the obtained data. The effectiveness is 86%, respectively, 90% compared to involved patients as can be seen in Figure 6.

**Figure 7:**
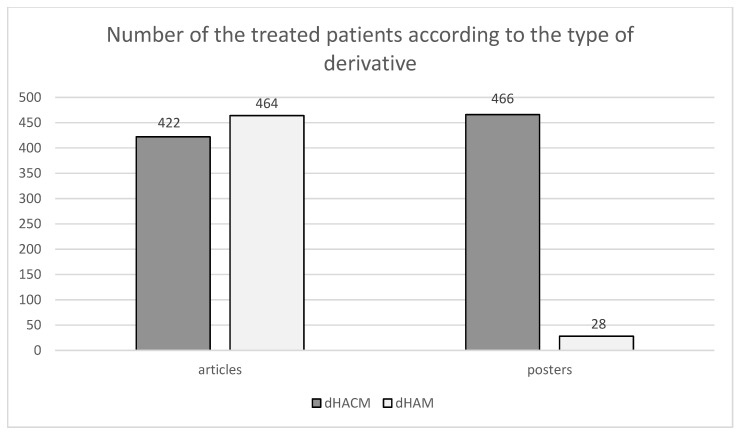
Shows the division of patients according to applied derivates (HAM/HACM). In the selected set of articles, 422 patients were given HACM and 464 patients were given dHAM. In the selected set of posters, dHACM was administered to 466 patients and dHAM was administered to 28 patients [1,2,3,4,5,6,7,8,9,10,11,12,13,14,15,16,17,18,19,20,21,22,23,24,25,26,27,28,29,30,31,32,33,34,35,36,37,38,39,40,41,42,43,44,45,46,47,48,49,50,51,52,53,54,55,56,57,58,59,60,61,62,63,64,65,66,67,68,69,70,71,72,73,74,75,76,77,78,79,80,81,82,83,84,85,86,87,88,89,90,91,92,93,94,95,96,97,98,99].

A comparable number can be observed in the articles of patients treated with amniochorionic membrane and amniotic membrane derivatives. However, in the case of posters, there is a considerable difference between treatment with the amniochorionic membrane and treatment with the amniotic membrane as can be seen in Figure 7.

Sources differ also in the frequency of the derivatives’ applications during the treatment. Several articles focused on the comparison of the group of patients who were administered derivatives each week and the group of patients who were administered derivatives every other week [23,24]. These articles conclude that the weekly application of derivatives supports the healing process more and the groups of patients healed faster than the groups of patients who were treated with one application in 14 days.

The research also shows that it is suitable to apply fresh derivatives or change applied derivatives several times during the treatment. Adequately planned reapplication of derivatives can lead to sooner healing. However, the optimal number of applications depends on the wound, its size, and the patient’s general condition.

Treatment with amniochorionic membrane derivatives can be used in a wide range of various injuries and illnesses. One of the most commonly treated conditions is wounds related to the advanced stage of diabetes and the occurrence of non-healing leg ulcers. Treatment of venous ulcers located in the lower limbs is typical for use of derivative applications and it is highly covered in the studies [23,25,26,27,28,29]. Other derivative applications are post-surgery non-healing wounds and other injuries. It can also be used in the treatment of burns of all degrees [10]. An interesting use is also in tympanoplasty where the amniochorionic membrane derivative enabled a patient’s faster hearing recovery [30]. It can further be used in dentistry [31] and ophthalmology [32].

An advantage of this treatment is also easy preparation of derivatives for wounds of different sizes. Application is not affected by wound size which means it can be used to treat small non-healing wounds no larger than 1 cm square, but also to treat large wounds larger than 100 cm^2^ [33].

In the processed set, there are also several articles that could not be processed by listing laid down parameters. This relates to several articles describing the general use and characteristics of therapeutic derivatives [31,34,35,36,37,38,39]. Several articles describe the course of treatment of chronic wounds with derivatives; however, the information contained is not supported with published data or they do not contain annotations to other studies [40,41,42,43,44,45]. Some of the studies do not define treatment effectiveness with the healing of the wound, but with other parameters, which complicates comparison across the whole set [25,46,47].

This section may be divided into subheadings. It should provide a concise and precise description of the experimental results, their interpretation as well as the experimental conclusions that can be drawn.

## 5. Discussion

Chronic wounds represent a serious and unpleasant health challenge for patients and clinicians. The availability of grafts of various sizes and shapes, which reduces the waste of graft material, also contributes to cost-effectiveness. Handling characteristics, long shelf life with no need for extensive graft preparation, all reduce bureaucratic and clinical time often associated with the utilization of advanced wound care products. Determining the cost-effectiveness of any advanced therapy or product in wound medicine requires consideration of many variables, including how many wounds heal completely and how quickly the wound closes. Although advanced wound care products can be expensive, costs can be reduced by shortened treatment, rates of complications, fewer hospitalizations, and lower rates of amputation [25,27].

Snyder et al., identified 76 commercially available skin substitutes to treat chronic wounds. The majority of these do not contain cells and are derived from the human placental membrane, animal tissue, or donated human dermis. Including noninfected and infected wound costs, the estimated cost of care for diabetic foot ulcers ranged from $6.2 billion to $18.7 billion, for venous leg ulcers the range was $0.7 billion to $1.5 billion, and for pressure ulcers the range was $3.9 billion to $22 billion [45,48,49].

From this research it follows that it is felicitous to do the application of fresh derivatives or possibly the replacement of the derivatives several times during the treatment. Well-planned reapplication of the derivatives can support wound healing and shorten the time of treatment. The optimal number of applications is quite versatile, it depends on the type of wound, its size, and the patient’s diagnosis. In addition, the wound must be well prepared before application, without infection, necrosis, and signs of inflammation.

Many studies proved that chronic wounds treatment with amniotic tissue derivatives is faster than the standard treatment. The time of treatment and number of applications depends on the individual parameters of the wound and varies in the sources. On average the time of healing does not exceed 10 weeks [23,26,50].

**Figure 8:**
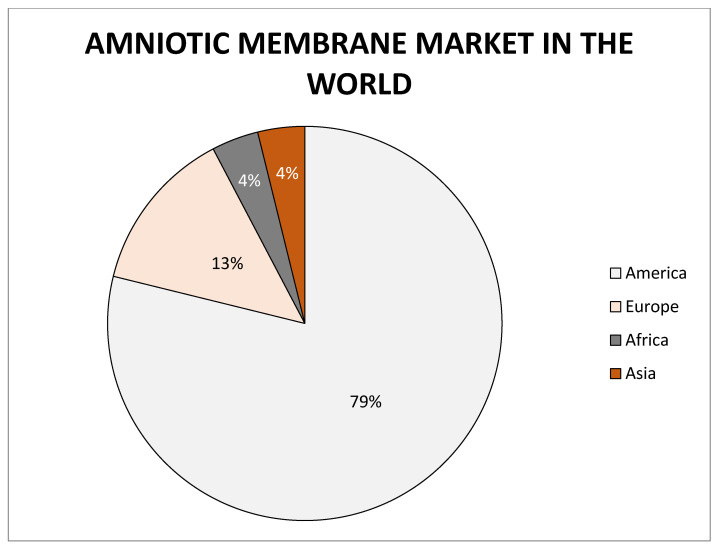
Representation of world market of amniotic membrane.

The quality and success of treatment in which placental derivatives are used also illustrates the information that company MiMedx has distributed of more than one million medicinal products [51]. Other dominating companies are Amnio Technology LLC, Celularity Inc., Human Regenerative Technologies LLC, Katena Products Inc., Integra LifeSciences Corporation, Skye Biologics Inc., and Amniox Medical Inc. The global amniotic membrane market size was valued at USD 2.26 billion in 2017 and the global amniotic membrane market size is expected to reach USD 5.81 billion by 2025, according to a report by the Grand View Research, Inc. Overall most companies focused on the research and development or manufacturing of amniotic membranes are located in the United States of America (Figure 8).

The amniotic membrane has gained popularity as a biologic dressing and/or scaffold for tissue regeneration. The amniotic membrane was used for numerous applications as a surgical dressing for burns and as an adjunctive tissue in surgical reconstruction of the oral cavity, bladder, and also for tympanoplasty, arthroplasty, the repair of omphaloceles, and prevention of adhesions in pelvic and abdominal surgery. All of these applications are the outcome of the interesting features of the amniotic membrane, for example its antimicrobial characteristic has made it a suitable choice for postsurgery applications in wound healing, burn injuries, dental injuries, and ophthalmology as bacterial infection and bio-film growth are regular in these sites [15,16].

The use of amniotic membranes or amniotic/amniochorion membranes has great potential in other medical fields also such as orthopedics (tendons, osteoarthrosis), gynecology (vaginoplasty), urology (using in radical prostatectomy), and others. Its usage has low ethical problems [16,30,52,53,54,55]. The potential of amniotic membrane use is also proved by 139 worldwide registered, continuing, or terminated clinical trials https://clinicaltrials.gov, accessed on 11 January 2021).

## Data Availability

Not applicable.
